# Evaluating patient perspectives of endovascular created arteriovenous fistulas for dialysis access (EndoAVF)

**DOI:** 10.1186/s12882-024-03475-4

**Published:** 2024-01-26

**Authors:** Melanie Field, A. Z. Khawaja, J. Ellis, R. G. Jones, N. G. Inston

**Affiliations:** 1 Department of Renal Transplantation and Dialysis Access Surgery, Queen Elizabeth Hospital, Edgbaston, University Hospitals Birmingham, Birmingham, West Midlands B15 2TH UK; 2 Department of Diagnostic and Interventional Radiology, Queen Elizabeth Hospital, Edgbaston, University Hospitals Birmingham, Birmingham, West Midlands UK

**Keywords:** Vascular access, End stage renal failure, Haemodialysis, Patient reported outcomes, Vascular access questionnaire, Endovascular arteriovenous fistula, Patient experience

## Abstract

**Background:**

Patient reported experience measures are contemporary quality indicators that focus on evaluation of healthcare delivery processes. While surgical arteriovenous fistulas (otherAVF) are preferred for haemodialysis vascular access, fears about surgery and complications often result in refusal/delays. A new technique of endovascular arteriovenous fistula creation (EndoAVF) has been developed and as part of it’s ongoing introduction into our unit, the patient perspective was felt critical to its evaluation. The Vascular Access Questionnaire (VAQ) provides a mechanism for identifying and scoring perceptions in this setting.

**Method:**

Patients who had previously undergone EndoAVF formation were approached to undertake the VAQ as part of a service evaluation of their experience. In addition to the components of the VAQ, data questions relating to the patient’s perception of their access were gathered. Results were compared with a matched historical cohort of surgically created fistulas (otherAVF) patients.

**Results:**

Patient satisfaction and self-reported ease of use with EndoAVF were high. Overall VAQ scores were similar between the EndoAVF and the surgically created cohort. Functionally, there was no significant difference in perception of their fistula by patients, irrespective of them being created surgically or radiologically.

**Conclusion:**

Although numbers in this report are small limiting exploration of preserved inherent heterogeneity, we provide a useful initial patient reported experience and perspectives on comparative functional use of radiologically and surgically created AVFs. As real world experience gathers, future larger cohorts with adequate sampling may allow exploration of patient reported experiences and outcome measures.

## Background

The first published report of an arteriovenous fistula (AVF) created for haemodialysis (HD) access was in 1965 by Brescia, Chimino, Appell and Hurwich [1]. Autologous vascular access (VA) is widely recognised as the “gold standard” for HD due to the association with lower morbidity, mortality, lower risk of hospital attendance and some evidence of better patient satisfaction [2]. However, the outcomes from surgical AVF formation and use are still not ideal. Concerns include primary failure and failure to mature and prolonged time to achieve functional maturation with stable two needle dialysis. Over the medium to longer term, AVFs can become problematic as can develop steno-occlusive disease which can result in thrombosis and also undergo aneurysmal development [3–5]. The difficulties with primary failure and failure to mature can lead to patients having to undergo multiple radiological or surgical procedural attempts to create or maintain a working native dialysis access. This can potentially lead to procedural fatigue and patients refusing further attempts through frustration, desire to avoid further attempts at surgery or radiological intervention and prioritizing what would be perceived as more relevant to daily living and well-being [6].

In the more than 50 years since their first description, the way that AVFs have been created has seen very little change and predominantly been under the remit of open vascular surgery. More recently advances in technology has allowed the advent of devices that have permitted these to be created through endovascular techniques, potentially opening the field to interventionalist with clinical experience in Interventional Radiology or Interventional Nephrology as well Vascular surgeons competent in interventional techniques.

Devices for the creation of these endovascular fistulas (EndoAVFs) currently commercially available include the WavelinQ (BD Medical, Franklin Lakes, NJ) and Ellipsys system (Medtronic, Dublin, IRL). The former is a 4 F dual catheter system which employs a single 0.7 s burst of radiofrequency energy for the creation of the AVF while the latter is a 6 F single catheter system which utilises pulsed thermal energy. Technical description of each system have been previous described and are coming up to having a decade of published evidence [7, 8]. In brief, both systems require specific anatomy of the proximal forearm deep radial, ulnar or interosseous vessels for the selection of an AVF creation site with the potential for shared fistularised venous outflow via deep and superficial systems of the upper arm achieved [9]. Similar to surgical AVFs, physiological maturation and selection of specific venous segments for cannulation and use for haemodialysis is based on clinical examination and ultrasound Doppler flow assessments as per well-established standard clinical examination practice by a trained dialysis nurse specialist [2, 10]. Early experience with these fistulas has shown them to be safe and efficacious and trials are ongoing to evaluate their longer-term outcomes (NCT04634916).

With respect to recommendations for procedural treatment and management, increasing emphasis is being attributed to the patients’ perspectives. This is being adopted across many different aspects of medicine and within our service we place a significant emphasis on the patient’s viewpoint for joint decision making with the patient being central to providing individualised patient tailored care. The Vascular Access Questionnaire (VAQ), described in 2008 by Quinn et al., consists of a patient-reported questionnaire composed of 17 VA related questions, which are scored on a 0–4 Likert scale [11]. We have shared our previous experience of service evaluation by capturing patient perspectives utilising VAQ [12]. As part of an evaluation into the role of endovascular fistulas within out service we aimed to examine the patient’s perspective on their EndoAVFs. Although some limited reports of capturing patient perspectives of their vascular access including AVFs have been published, to the best of our knowledge, application of the VAQ in patients with EndoAVFs has not been previously published.

## Methods

Patients who had previously undergone an EndoAVF formation and were undergoing HD were approached to take part as part of service quality improvement and feedback initiative. Data relating to cannulation technique was not specifically captured but the predominant cannulation technique in our centre is ropeladder or area cannulation. All EndoAVFs were initiated and needled following the same standard cannulation pathways followed by the surgical cohort of patients employing standard sharp needles with the aim of developing ropeladder cannulation tracts.

The overall aim was to capture the patient’s perspectives of their EndoAVF and how this could compare to previously captured perspectives of surgically created fistulas [12].

Data captured included patient and their HD vascular access specific demographics, perspective on the creation procedure and overall satisfaction with the fistula including open ended questions and free text comments. Due to the restrictions imposed by the Covid pandemic the questionnaire was administered by telephone. Data was collated and managed using REDCap electronic data capture instrument hosted at the University of Birmingham and exported for analysis to IBM SPSS version 22 (IBM Corp. Armonk, NY), and GraphPad Prism version 7.0 (GraphPad Software, San Diego, Calif. USA) [13]. The study was registered with the audit committee at our institution as a service evaluation (CARMS-16,320).

### Reference cohort

In addition to the patients with EndoAVF cohort, a reference cohort of patients with other types of VA at our centres was also available. This reference cohort originally comprised *N* = 749 patients, and details of the demographics and data collection have been reported previously [12]. Of these, the subset of *N* = 210 patients with grafts or tunnelled lines for access were excluded, to leave only those with functional and in use AVFs. In addition, since the longest duration of endovascular access in the EndoAVF cohort was five years, patients whose current access had a greater duration than this (or where the duration was not stated) were excluded from the reference cohort (*N* = 208). The remaining *N* = 331 comprised the “OtherAVF” cohort.

### Statistical methods

Comparisons between the EndoAVF and OtherAVF cohorts were performed using Fisher’s exact tests for nominal variables, and Mann-Whitney U tests for ordinal or continuous variables. Continuous demographic variables are reported as medians and interquartile ranges (IQRs), with the VAQ score additionally summarised using means. For all analyses, *p* < 0.05 was deemed to be indicative of statistical significance.

## Results

### Cohort characteristics

From the cohort of the first 25 patients who were potentially approachable (creation more than 2 months ago) 16 responses were collected, 2 patients had died, 2 refused to participate and 5 had primary failure following creation and were therefore not eligible. The questionnaire was completed by the *N* = 16 patients, the demographics of whom are reported in Table 1. These patients had a median age of 61 years (IQR: 45–67), with the majority being male (75%) and of Asian (50%) or White (44%) ethnicity.

**Table 1:** Demographic characteristics of included cohorts of patients with endovascular and matched surgically created arteriovenous fistula

	EndoAVF		OtherAVF		
	N	Statistic	N	Statistic	*p*-Value
Age (Years)	16	61 (45–67)	331	65 (55–75)	0.056
Gender (% Male)	16	12 (75%)	331	198 (60%)	0.298
Ethnicity	16		330		0.393
*White*		7 (44%)		167 (51%)	
*Asian*		8 (50%)		110 (33%)	
*Black*		1 (6%)		52 (16%)	
*Mixed*		0 (0%)		1 (0%)	
Peripheral Vascular Disease	16	0 (0%)	331	36 (11%)	0.390
Diabetes	16		331		0.209
*No*		10 (63%)		198 (60%)	
*Diet Controlled*		0 (0%)		27 (8%)	
*Tablet Controlled*		3 (19%)		21 (6%)	
*Insulin*		3 (19%)		85 (26%)	
Heart Problems	16	5 (31%)	331	99 (30%)	1.000
Duration of Haemodialysis (Years)	16	3 (2–4)	331	2 (1–4)	0.152
Duration of Current Access (Years)	16	2 (2–4)	331	2 (1–3)	0.154
Current Access Type	16		328		*N/A*
*Endovascular AVF*		13 (81%)		0 (0%)	
*Brachiocephalic AVF*		2 (13%)		177 (54%)	
*Brachiobasilic AVF*		0 (0%)		35 (11%)	
*Radiocephalic AVF*		0 (0%)		116 (35%)	
*Tunnelled Line*		1 (6%)		0 (0%)	
Any Previous Access**	16	4 (25%)	331	57 (17%)	0.497
Any Radiological Intervention**	15*	4 (27%)	331	89 (27%)	1.000
Aneurysmal Fistulas	15*	5 (33%)	331	65 (20%)	0.197

Data are reported as N (column %), with *p*-values from Fisher’s exact tests, or as median (interquartile range), with *p*-values from Mann-Whitney U tests. Bold *p*-values are significant at *p* < 0.05. *Excludes *N* = 1 from the EndoAVF group, who was using a tunnelled line at the time of the questionnaire. **Within the last 12 months (and excluding the initial procedure)

At the time of the questionnaire, *N* = 3 were not using their endovascular fistulas, with *N* = 2 instead using brachiocephalic fistulas, and *N* = 1 using a tunnelled line.

In addition to the EndoAVF cohort, data was also available for a reference cohort of *N* = 331 patients receiving other types of AVF (OtherAVF cohort), namely brachiocephalic (54%), radiocephalic (35%) and brachiobasilic (11%). The characteristics of this cohort were largely comparable to the EndoAVF cohort.

### VAQ score

Since the questionnaire asked patients for views on their current access, responses to these questions in the EndoAVF cohort were only meaningful for the *N* = 13 who were using their fistulas at the time of the questionnaire. These patients had a median VAQ score of 4 (IQR: 1–7), which did not differ significantly from the 3 (1–7) in the OtherAVF cohort (mean: 4.5 vs. 5.2, *p* = 0.915). However, analysis of the individual components of the VAQ score (Table 2) found the EndoAVF cohort to have a significantly elevated pain score compared to the OtherAVF cohort (mean 0.46 vs. 0.28, *p* = 0.040). Pain relates to the overall assessment of pain relating to any aspect of the patients current AVF and does not specifically relate to cannulation or other potential causes of discomfort such, as post cannulation or infiltration haematoma.

**Table 2:** Components of the Vascular Access Questionnaire with respective scores and comparison between cohorts of endovascular and surgically created arteriovenous fistulas with majority denoting no significant difference as perceived by patients apart from cannulation pain during early use

VAQ Component	Cohort	Mean	Proportion of Patients Scoring:					*p*-Value
			0	1	2	3	4	
Bleeding	EndoAVF	0.31	77%	15%	8%	0%	0%	0.518
	OtherAVF	0.25	84%	11%	2%	3%	1%	
Pain	EndoAVF	0.46	62%	31%	8%	0%	0%	**0.040**
	OtherAVF	0.28	85%	8%	1%	5%	1%	
Bruising	EndoAVF	0.38	77%	15%	0%	8%	0%	0.907
	OtherAVF	0.34	78%	13%	5%	3%	1%	
Swelling	EndoAVF	0.00	100%	0%	0%	0%	0%	0.161
	OtherAVF	0.20	87%	8%	3%	2%	0%	
Redness	EndoAVF	0.00	100%	0%	0%	0%	0%	0.597
	OtherAVF	0.02	98%	2%	0%	0%	0%	
Infection	EndoAVF	0.00	100%	0%	0%	0%	0%	0.843
	OtherAVF	0.00	100%	0%	0%	0%	0%	
Clotting	EndoAVF	0.31	85%	0%	15%	0%	0%	0.565
	OtherAVF	0.18	89%	6%	2%	2%	0%	
Appearance	EndoAVF	0.23	85%	8%	8%	0%	0%	0.784
	OtherAVF	0.35	82%	8%	3%	5%	2%	
Worries Working Well	EndoAVF	0.62	69%	15%	8%	0%	8%	0.773
	OtherAVF	0.43	71%	19%	6%	3%	1%	
Attending Dialysis Early	EndoAVF	0.00	100%	0%	0%	0%	0%	0.197
	OtherAVF	0.20	89%	6%	3%	3%	0%	
Leaving Dialysis Late	EndoAVF	0.00	100%	0%	0%	0%	0%	0.061
	OtherAVF	0.41	78%	10%	5%	5%	1%	
Problems Sleeping	EndoAVF	0.92	62%	15%	0%	15%	8%	0.593
	OtherAVF	0.67	66%	14%	8%	11%	2%	
Protecting Your Access	EndoAVF	0.23	92%	0%	0%	8%	0%	0.120
	OtherAVF	0.49	70%	17%	7%	5%	1%	
Interfering with ADL	EndoAVF	0.08	92%	8%	0%	0%	0%	0.430
	OtherAVF	0.25	85%	8%	4%	2%	1%	
Interfering Leisure Activities	EndoAVF	0.15	85%	15%	0%	0%	0%	0.885
	OtherAVF	0.26	84%	8%	5%	2%	0%	
Worries About Hospitalisation	EndoAVF	0.23	92%	0%	0%	8%	0%	0.349
	OtherAVF	0.35	81%	9%	6%	2%	2%	
Worries How Long Access Will Last	EndoAVF	0.62	54%	31%	15%	0%	0%	0.394
	OtherAVF	0.54	68%	19%	7%	4%	2%	

Results are based on *N* = 13 vs. *N* = 331 from the EndoAVF vs. OtherAVF cohorts. Data are reported as mean scores, and the proportion scoring each number of points: 0 = Not at All, 1 = A Little, 2 = Moderately, 3 = Quite a Bit, 4 = Extremely. *p*-Values are from Mann-Whitney U tests, and bold p-values are significant at *p* < 0.05

### Satisfaction with endovascular fistulas

All patients reported their EndoAVFs to be somewhat/very easy to use, were somewhat/very satisfied with their fistulas and, consequently, stated that they would recommend their current access. The distributions of responses to these questions demonstrated non-inferiority in the EndoAVF patient cohort as they were similar to the OtherAVF cohort (Table 3satisfaction domains all *p* > 0.05).

**Table 3:** Patient reported satisfaction with their native access as compared across cohorts of endovascular and surgically created arteriovenous fistulas demonstrating high levels of satisfaction across all domains and no significant diference in perception

	EndoAVF	OtherAVF	*p*-Value
Is your access easy to use?			0.527
*Very Difficult*	0 (0%)	1 (0%)	
*Somewhat Difficult*	0 (0%)	20 (6%)	
*Somewhat Easy*	2 (15%)	51 (15%)	
*Very Easy*	11 (85%)	258 (78%)	
How satisfied are you with your current access?			0.773
*Very Dissatisfied*	0 (0%)	2 (1%)	
*Somewhat Dissatisfied*	0 (0%)	4 (1%)	
*Somewhat Satisfied*	1 (8%)	27 (8%)	
*Very Satisfied*	12 (92%)	297 (90%)	
Would you recommend your current access?			0.274
*No*	0 (0%)	8 (2%)	
*Maybe Not*	0 (0%)	3 (1%)	
*Maybe*	0 (0%)	17 (5%)	
*Yes*	13 (100%)	302 (92%)	

Results are based on *N* = 13 vs. *N* = 330 from the EndoAVF vs. OtherAVF cohorts. Data are reported as N (column %), with p-Values from Mann-Whitney U tests, and bold *p*-values are significant at *p* < 0.05

### Free comment analysis

As part of the questionnaire, open ended questions were included to determine how the patients found initial and current cannulation, thoughts on comparison with surgically created fistulas and how they would feel about future access or advise others (*n* = 6 gave free comments).

- Patients specifically commented that their EndoAVFs had a better appearance than other fistulas in their units.
- Specifically mentioned they would want another EndoAVF for preference in the future.
- Patients with experience of a previous surgical fistula creation specifically mentioned that the process of formation for their EndoAVF was less uncomfortable than surgical fistula creation (different anaesthetic protocols exist for each procedure and may contribute to this).

From a cannulation perspective 5 patients mentioned that there was some initial hesitation from the dialysis nursing staff with respect to initial cannulation, but all felt that cannulation was now without problem.

## Discussion

To the authors knowledge this is the first evaluation of endovascular created fistulas from the patient perspective utilising the Vascular Access Questionnaire. Whilst the numbers are small, reflecting the relative recent introduction and early use of fistulas created with this technique, they provide an extremely useful initial insight into the perspectives of patients who have undergone the procedure and capturing their experiences of their functional use.

Within our unit initial concerns raised in the Dialysis Vascular Access community about difficulties with cannulation or achieving stable functional maturation of the EndoAVF have not necessarily been proven in the medium term. Patients reported that once cannulation was established, their EndoAVFs were easy to use. Overall the EndoAVFs had very high satisfaction scores with none of the patients dissatisfied with their access. Overall, they all would recommend it and consequently could denote non-inferiority to other surgically created AVFs. A longitudinal comparative comparison during early use may be more suitably placed to capture challenges during initiation of cannulation.

On analysis between the two groups, the components of the VAQ were very similar between the groups. It is possible that this may reflect the small sample size within the EndoAVF group and therefore the difficulty in reaching statistical significance. Future larger cohorts with adequate sample sizes may allow exploration of inherent and any possible heterogeneities across cohorts. One of the differences that was significant between the two groups were pain scores, with these being higher amongst the EndoAVF group. Although statistically significantly the difference between the groups was small (mean 0.46 vs. 0.28) and overall the pain scores were both very low for either group indicating that pain was not a major concern. A caveat to acknowledge could be the potential for learning curves associated with differences in clinical examination of “softer” shared flow EndoAVF versus higher flow surgical AVFs, which is usually the main stay of assessment prior to cannulation of any AVF in the majority of centres around the world. As our centre has been involved with the creation and use of EndoAVFs over the last 7 years, this aspect may perhaps be a missed opportunity to capture important reflections from staff when evaluating nurses/staff’s perspectives of initiating an EndoAVF service, and maybe better placed with newer centres in their early journey of using the technology [14].

Whilst both otherAVF and EndoAVF have very high satisfaction with patients there may be an advantage to patients who will no longer attempt a further surgically created access as an EndoAVF is created radiologically, possibly providing an alternative method or technology as a choice for the patient. Similarly, support for EndoAVF was universal amongst the patients who had undergone formation with 100% stating they would recommend whereas support was less emphatic for otherAVF, although again a larger cohort need may be considered a caveat for discussion of experiences. A functioning easy to cannulate fistula was the desired end result as captured from these patient perspectives both from the EndoAVf and surgical AVF cohort.

Both groups VAQ scores provide a snapshot of patients’ perspectives on their access and this is extremely useful and albeit small numbers, provides the best patient reported evidence we currently have. Further development of a specific or tailored PROMs and patient experience tools, for example the VASQoL, may provide an opportunity to examine patients’ perspectives longitudinally at different timepoints across a patients vascular access journey [15].

## Conclusion

Although numbers in this study are small, we provide a useful initial patient reported experience and perspective on comparative functional use of endovascular and surgically created AVFs. Functionally, there was no significant difference in perception of a fistula by patients irrespective of them being created surgically or radiologically– “a fistula is a fistula and cannulation is cannulation”. As a new and evolving technology this may prove an extremely useful addition, potentially increasing options available for sites of vascular access and additionally opening the field to interventional colleagues. Follow through to functional use after creation is an important step and patients report both high satisfaction for EndoAVFs and traditional surgically created arteriovenous fistulas.

## Data Availability

The datasets used and/or analysed during this current study are available from the corresponding author on reasonable request.
